# Associations among Food Security, School Meal Participation, and Students’ Diet Quality in the First School Nutrition and Meal Cost Study

**DOI:** 10.3390/nu13020307

**Published:** 2021-01-22

**Authors:** Sarah Forrestal, Elizabeth Potamites, Joanne Guthrie, Nora Paxton

**Affiliations:** 1 Mathematica, 111 E Wacker Drive, Suite 3000, Chicago, IL 60601, USA; lpotamites@mathematica-mpr.com; 2 USDA Economic Research Service, 355 E Street SW, Washington, DC 20024, USA; joanne.guthrie@usda.gov; 3 Mathematica, 600 Alexander Park, Suite 100, Princeton, NJ 08540, USA; npaxton@mathematica-mpr.com

**Keywords:** food security, National School Lunch Program, School Breakfast Program, free and reduced-price lunch, energy intakes, Healthy Eating Index, school-age children, School Nutrition and Meal Cost Study, school nutrition dietary assessment study

## Abstract

The Healthy, Hunger-Free Kids Act of 2010 updated the nutrition standards in the National School Lunch and School Breakfast Programs (NSLP and SBP) and expanded universal free meals’ availability in low-income schools. Past studies have shown that school meals are an important resource for children in food-insecure households. This analysis used data from the School Nutrition and Meal Cost Study to classify students as food insecure (FI), marginally secure (MS), or food secure (FS). Diet quality from school and nonschool foods that students consumed was assessed using Healthy Eating Index (HEI)-2010 scores. Chi-squared and two-tailed *t*-tests were conducted to compare school meal participation, students’ energy intakes, and diet quality across food security groups. FI and MS students were significantly more likely to participate in NSLP than FS students (79%, 71%, and 49%, respectively). SBP participation followed a similar pattern but was lower (38% FI, 33% MS, and 16% FS). Compared to FS students, FI and MS students more likely attended schools offering SBP, universal free meals, or afterschool snacks and suppers. School meals contributed significantly more energy to FI and MS students’ diets than to FS students (22%, 20%, and 13%, respectively). All groups’ dietary intakes from school foods were of higher quality than non-school foods. These findings highlight the role of school meals in meeting the energy and diet quality needs of FI and MS students.

## 1. Introduction

The U.S. Department of Agriculture (USDA)’s National School Lunch Program (NSLP) and School Breakfast Program (SBP) are integral parts of the U.S. federal government’s food assistance and nutrition safety net. Through these programs, participating schools serve lunches and breakfasts meeting federally established nutrition standards and receive reimbursements for meal costs from USDA that vary based on the household income of participating children. Children from families with incomes at or below 130% of federal poverty guidelines can receive meals for free; those whose family income is between 130% and 185% of poverty pay a reduced price [1]. (In 2015, the federal poverty guideline was an annual income of $24,250 for a family of four [2].) Children are certified to receive FRP school meals by either submitting an application or being directly certified because their households participate in other federal assistance programs [1]. USDA provides higher levels of reimbursements for free and reduced-price NSLP and SBP meals. Children from higher-income families pay what is called “full price”, although USDA still partially subsidizes their meals. Regulations require that all children receive the same meal and differences in payment status should not be identifiable.

In 2019, the NSLP served 29.4 million children on a typical school day, and the SBP served close to 15 million children [3]. Almost all public schools participate in the programs, and meals are available to all attending students. However, 74% of lunches and 85% of breakfasts were served free or reduced-price (FRP) to low-income students in 2019.

In 2010, Congress passed the Healthy, Hunger-Free Kids Act (HHFKA) [4]. It mandated updated nutrition standards in line with federal dietary guidance, including age-based calorie minimums and maximums; required inclusion of whole grain-rich foods; a wider range of vegetables, with dark green and red/orange vegetables and legumes required to be served weekly; more whole fruit; and low or nonfat milk as the standard milk offered. Implementation of menu changes began in the 2012–2013 school year (SY) [5].

The HHFKA also established a new policy, the Community Eligibility Provision (CEP), under which schools serving primarily low-income children could offer meals to all students at no cost—a policy referred to as “universal free meals”. Previously, some schools offered universal free meals through other USDA provisions, but the CEP considerably expanded the number of schools offering them. The CEP required participating schools to offer the SBP, increasing availability of school breakfasts. The CEP option began as a pilot in selected states and was implemented in all states by SY 2014–2015 [6]. Finally, the HHFKA established availability of afterschool suppers as part of afterschool programs in low-income areas.

These changes could be expected to have important impacts on the diets of children living in households that struggle with food insecurity. Food security is defined as access by all people at all times to enough food for an active, healthy life [7]. Households unable to acquire adequate food for one or more household members because of insufficient money and other resources for food are considered food insecure [8]. In 2019, 14% of households with children were classified as food insecure (adults, children, or both were food insecure) at some time during the year [7]. Parents and other household adults typically try to shield children from experiencing food insecurity; nevertheless, children were reported to be food insecure in 7% of households with children [7].

Food insecurity among children is associated with many adverse impacts on physical and mental health, as well as negative social and academic outcomes [8,9]. Children living in food-insecure households have been reported to have poorer overall health, have increased frequency of chronic health conditions and more hospitalizations, and be more likely to be iron-deficient, and male children have been found to have lower bone density, and they are more likely to experience psychological distress, including higher rates of depression and anxiety and, among adolescents, suicidal ideation [8,9]. Behavior problems and academic challenges, including slower progress in math and reading and higher likelihood of repeating a grade among elementary-age children, are also associated with food insecurity [8,9].

Previous research has shown school meals to be important resources for children in food-insecure households. In an analysis of data from the USDA’s School Nutrition Dietary Assessment III (SNDA-III), conducted in SY 2004–2005, Potamites and Gordon found that children from food-insecure households were more likely to participate in school meal programs and obtained a larger share of their food and nutrient intakes from school meals than other children [10]. Using national food consumption survey data, Smith found that school meals provided the highest nutritional quality contribution to the diets of children from food-insecure households compared to food obtained at home or elsewhere, with quality measured by the USDA’s Healthy Eating Index (HEI) [11]. Both studies were completed before the HHFKA-driven changes in school meals. Post HHFKA, substituting school meals for meals from home was associated with higher nutritional quality for students across the income distribution [12]. The more widespread availability of school breakfasts and afterschool suppers may have increased the importance of school food in the diets of food-insecure children, whereas changes in the nutrition standards may have impacted its contribution to intakes of nutrients and important food groups for all students.

The objective of this paper is to examine the association between food security status, school meal participation, and students’ diet using data from the first School Nutrition and Meal Cost Study (SNMCS-I), a comprehensive, national data set collected following the implementation of HHFKA-mandated changes. SNMCS-I is unique in that it links student-reported dietary intakes at school with school-reported menus to identify the contributions of school meals to students’ diets. We hypothesized that, compared to food secure (FS) students, food insecure (FI) students and, to a lesser extent, marginally secure (MS) students make more use of the USDA school meal programs, and that these programs contribute more to their diet in terms of both quantity and quality, as assessed by calories and HEI scores, respectively.

## 2. Materials and Methods

### 2.1. Study Population and Data Collection Methods

SNMCS-I was a national study of public, noncharter schools that participated in NSLP in SY 2014–2015 and the diets of students attending those schools. First, SFAs were randomly selected, then schools were randomly selected within the SFAs, and then students were randomly selected within schools. The samples were selected to be nationally representative at each level. SFA directors and school principals were contacted and recruited to participate. After a school agreed to participate, school staff submitted a student roster for sampling. The parents/guardians (hereafter referred to as parents) of sampled students were mailed information about the study and invited to participate. Parents provided consent and students provided assent. Most data were collected during one scheduled week, called the “target week,” in each SFA.

An overview of the study and detailed methodological information are reported elsewhere [13,14]. The New England Institutional Review Board reviewed and approved the study protocol (approval number 120160196).

#### 2.1.1. Student and Parent Data Collection

The Child/Youth and Parent Interviews captured information about school meal participation, student and household demographics, participation in other nutrition assistance programs, and food security over the past year using the USDA’s 18-item U.S. Household Food Security Survey Module [15]. Students were interviewed during the target week. The interview procedures differed by school type. Middle and high school students completed the interviewer-administered Child/Youth Interview and 24-h dietary recall about the prior school day. They reported intakes from midnight to midnight in the prior day, called the “target day”. They received a $15 gift card for completing the interview on a weekday or a $20 gift card for completing the interview on a Saturday to report what they ate on Friday. Their parents completed the Parent Interview by telephone after the students were interviewed and were mailed a $15 check afterward.

In contrast, the target day was the same as the interview day for elementary school students. They completed the Child/Youth Interview and a portion of the recall shortly after lunch to report everything they ate and drank since waking through lunch on the interview day. Interviewers gave elementary school students a nonquantitative food diary to use at home, and the students and their parents together completed the rest of the recall interview in person the next day to report intakes from after lunch to midnight of the target day. The parents also completed the Parent Interview in person. Elementary school students were given a $5 gift card and their parents were given a $30 gift card for participating.

A total of 5033 students and their parents were contacted about the study, and 4141 agreed to participate. Of those, 3591 students and parents were attempted for data collection; 2165 students completed the Child/Youth Interview and dietary recall, and of those students, 1843 parents completed the Parent Interview and the food security module. The final weighted response rate for student–parent dyads is 89% [14].

#### 2.1.2. SFA and School Data Collection

The Menu Survey collected details about every food item and recipe so that school meal foods could be coded for nutrient analyses. School nutrition managers recorded information about all the foods offered in reimbursable school meals during the target week. They were mailed a $50 check after completing the Menu Survey. SFA directors and principals completed web surveys after the target week that captured information about school food service characteristics. The final weighted response rates for the Menu Survey, SFA Director Survey, and Principal Survey are 96%, 96%, and 87%, respectively [13].

### 2.2. Key Variables

#### 2.2.1. Student, Household, and School Characteristics

The student and household characteristics included in this study are child age, gender, and race/ethnicity, and household poverty level (the ratio of annual household income to the federal poverty guideline for the household size). Student certification for FRP meals was determined using the schools’ reimbursable meal sale data, which includes FRP certification status. If the data were missing, parent self-report of certification status was used instead. The school characteristics included whether the school participated in SBP, offered universal free meals (which includes CEP), or offered reimbursable afterschool meals or snacks.

The items in the food security module create three measures of household access to food over the past year. The three related scales measure the degree to which financial constraints affect the diets of all children in the household (the child scale), adults in the household (the adult scale), and the entire household (the household scale). Following the classification used by Potamites and Gordon [10], we classified students as insecure, marginal, or secure based on the adult food security measure. The reason for this is that household and child food security measures are sensitive to the age of the eldest child, because household food shortages are more likely to be reported as having affected a child in the household if at least one child is a teenager [16]. Therefore, in any analysis using the child or household food security scale, it is important to control for the age of the oldest child. SNMCS-I did not collect this information; therefore, we used the adult food security scale. For brevity in the text, we refer to students from households experiencing adult food insecurity as FI students.

#### 2.2.2. Identifying School Meal Foods and School Meal Participation

Foods and beverages were identified as a school meal food (that is, an item served as part of a reimbursable breakfast or lunch) if a student-reported item matched an item reported in the Menu Survey. Schools’ reimbursable meal sale data were the primary source of information about whether students participated in NSLP or SBP on the target day. For the 9% to 13% of students missing these administrative data for NSLP and SBP, respectively, what students reported eating was used to determine whether they participated in one or both school meals. Students were classified as NSLP participants if they reported eating at least three of the five required meal components and the components were on the lunch menu, or they reported eating at least one of the components and also said they ate a school lunch on the target day. Students were classified as SBP participants if they reported eating at least one of the three required meal components, the item was on the breakfast menu, and they said they ate a school breakfast on the target day [14].

#### 2.2.3. Healthy Eating Index-2010 Scores

School meal foods reported in the Menu Survey and dietary recalls were linked to energy and nutrient values in the 2011–2012 Food and Nutrient Database for Dietary Studies [17]. HEI-2010 scores were computed for both the school foods offered to students and the items students consumed. HEI-2010 consists of 12 component scores, calculated on a 1000-calorie basis, which are combined to produce an overall measure of diet quality [18,19]. As shown in Table 1, nine components assess the adequacy of the diet in meeting nutritional needs, and three components assess factors whose consumption should be moderated. The overall score range is 0 to 100. Component scores are presented as percentage of the maximum score for that component for ease of comparability across components with different maximum scores.

### 2.3. Analyses

Students with a completed dietary recall and Parent Interview with a completed food security module (*n* = 1843) were included in the analyses, with each student categorized into one of three groups: FI, MS, or FS. The groups were compared on their demographic and school characteristics, their NSLP and SBP participation rates, and their dietary intakes at and away from school. Statistical significance (*p* < 0.05) was assessed using two-tailed *t*-tests for continuous characteristics and Pearson’s chi-squared tests for proportions [21]. Using SAS 9.4 and Stata 16.1, point estimates, confidence intervals, and statistical tests accounted for the complex survey design and used weights that adjust for nonresponse. Group comparisons are purely descriptive; no causal claims are being made. Observed differences across groups do not control for the average characteristics of the groups in any of the findings. The results presented describe the average actual experience of each group, and do not attempt to equalize groups on all observed characteristics except for food security. For example, we purposefully did not control for race/ethnicity or income when describing the differences (or lack of differences) in participation rates or diet quality. These characteristics are closely intertwined with food security and our goal is provide useful descriptive statistics, not isolate a causal “effect” of food insecurity.

## 3. Results

### 3.1. Student, Household, and School Characteristics

FS students were significantly more likely than both FI and MS students to be white, less likely to be Hispanic, less likely to be in the lower-income groups, and less likely to be certified for FRP meals (Table 2). The FI and MS groups did not differ substantially except that the FI students were more likely to be at the lowest income. Both FI and MS students were more likely to attend schools offering SBP, universal free meals, and reimbursable snacks or suppers. All three groups attended schools offering meals of similar nutritional quality.

### 3.2. NSLP and SBP Participation

On the target day, significantly more FI and MS students than FS students participated in NSLP (Table 2). Although the pattern was the same for SBP, participation was much lower for all groups attending SBP-participating schools. Characteristics related to food access and availability—FRP certification and enrollment at schools offering universal free meals or afterschool snacks or suppers—were associated with higher NSLP and SBP participation (Table 3).

FRP-certified FI students were significantly more likely to participate in NSLP than noncertified FI students (Table 3). Because three-fourths of students who attended a school with universal free lunches were also FRP certified, it is not surprising to observe similarly high NSLP participation rates for FRP-certified students and those at a school with free lunch. The availability of universal free breakfast was also associated with substantially higher rates of SBP participation compared to FRP certification. The availability of reimbursable afterschool snacks and suppers was associated with higher NSLP and SBP participation among all students, but the differences were statistically significant only for FS students. Most FS students who attended a school that offered reimbursable afterschool snacks and suppers participated in NSLP, compared to a minority of FS students who attended a school that did not.

### 3.3. Percentage of 24-h Energy Intakes from NSLP and SBP Foods

To assess the contribution of school meals to the diets of FI and MS students, Table 4 presents the average percentage of calories students received from school meal foods. As expected, given the lower school meal participation among FS students, school meal foods contributed a smaller share of FS students’ total energy intakes compared to the intakes of FI and MS students. The differences in the percentage of energy from school meals were small across food security groups among students who participated in either NSLP or SBP, or both. These small differences may be due to more FI and MS students participating in both meals (Table 2).

### 3.4. Diet Quality of School Meal and Non-School Meal Foods Consumed by Participants

**Figure 1:**
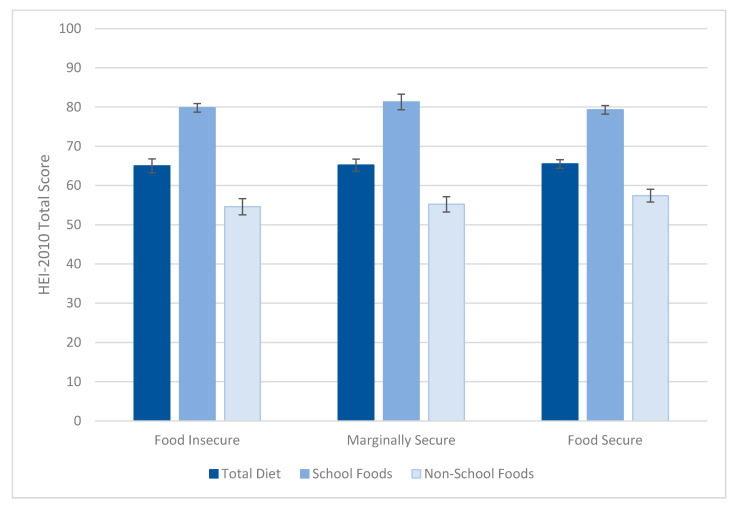
School meal participants’ diet quality (HEI-2010 scores), by food security status. Food security status was based on the adult food security measure.

The three groups did not differ in overall diet quality, and all groups’ dietary intakes from school foods were of higher dietary quality than the non-school foods they consumed (Figure 1). The quality of what students consumed from school meals was similar to the quality offered (Table 2). Table 5 and Table 6 present the mean percentage of maximum HEI-2010 total and component scores for foods from school meals and all other foods, respectively, for participants in at least one school meal program. The school meal foods component scores for total and whole fruit, whole grains, dairy, reduced refined grains, and empty calories were at the maximum or near maximum of the measure for all groups. The total protein score was at the near maximum for both school meal foods and non-school meal foods.

The only component for which nonschool meal foods are notably healthier than school meal foods is seafood and plant protein, possibly because students are not as frequently offered peanut butter or similar foods in school meals [14]. Both school meal and nonschool meal foods are under 50% of the maximum on the total vegetables, greens and beans, and reduced sodium level component scores.

FS students scored higher on the dairy component of nonschool foods than FI students and lower on the fatty acid component. Otherwise, no significant differences were found in the dietary quality of nonschool foods across the three food security groups. No significant differences were observed across the groups for any component of the school meal foods.

## 4. Discussion

This study found that FI and MS students were more likely to attend schools that participated in SBP, offered universal free meals, and offered reimbursable afterschool snacks or suppers, which suggests that the efforts to encourage participation in healthy school meals appropriately focused on students with greater need. Although FI and MS students were more likely to participate in school meals, which is consistent with the first hypothesis, participation was not universal; only 38% of FI and 33% of MS students in SBP-participating schools participated in SBP. This is scarcely different from participation levels in SNDA-III (37% and 26% for FI and MS students in SBP-participating schools, respectively), despite a 10 percent increase in SBP availability in the decade between studies [10]. The availability of SBP therefore does not seem to be sufficient to increase participation, although causality cannot be inferred. Instead, SBP participation was significantly higher in schools offering universal free meals or afterschool snacks or suppers. These policy options may decrease any stigma associated with participation or may be an indicator of an overall school culture that encourages participation. Strategies to make school breakfast more convenient or appealing to children, such as serving breakfast in the classroom at the beginning of the school day, rather than in the cafeteria before school starts, or offering “second-chance breakfasts” at mid-morning to late-arriving students have also been suggested to help increase participation [22,23]; further exploration of factors influencing SBP participation, particularly in schools with large proportions of students vulnerable to food insecurity, may provide more evidence of their effectiveness and identify other factors influencing participation.

School meals contributed significantly more to FI and MS students’ energy intakes, which reflects their higher rates of participation and is consistent with the hypothesis that school meal programs are an important contributor to diet quantity (that is, calories). Among participants, differences across food security groups were not statistically significant. In SNDA-III, school meals contributed significantly more energy to FI and MS participants’ diets than to FS participants’ diets; school meals made up 32% of the daily energy intake of FS participants, 30% of MS participants’ intake, and 27% of FS participants’ intake [10]. Differences between the studies may reflect the calorie maximums in the updated nutrition standards. However, the two studies used different methods to identify school meal participants. SNDA-III identification was based on reported intakes rather than administrative data, although the methods were compared using SNMCS-I data and only small differences in classification were found [14]. One might expect to see greater differences in the contributions of school meals to students’ energy intakes in SNMCS-I because participation was not based on whether any school meal foods were consumed, and FI and MS students may be more likely to consume those foods if their access to food outside of school is limited. It is unclear why differences were observed in SNDA-III but not SNMCS-I.

Analysis of the USDA’s National Food Acquisition and Purchasing Study (FoodAPS) found that FI households buy less food than other households of equivalent composition and, in particular, acquired less total fruit, whole fruit, total protein, and seafood and plant proteins compared to food-secure households, on a per-1000-calorie basis [24]. When analyzing FoodAPS data, Kuhn found that school meal programs played an important role in reducing “consumption crunches” (monthly cycles of reduced energy intakes associated with food assistance benefits running out) in low-income families with children during periods of reduced food expenditures [25]. This effect benefited both children and adults in the households, probably because the children’s ability to acquire food at school reduced strain on overall household food resources. Our finding that FI and MS children were more likely to participate in school meals and therefore acquire a larger share of their calories from these programs is consistent with Kuhn’s findings, and it indicates the important role these programs can play in supporting dietary adequacy for children in FI households.

Using data from the National Health and Nutrition Examination Surveys (NHANES) before implementation of the updated nutrition standards, Smith found that consumption of foods obtained at school improved the quality of diets of low-income and FI students as measured by the HEI, but it did not improve diet quality for higher-income children [11]. However, a similar analysis conducted with NHANES data collected postimplementation found improvements in students’ diet quality across all income groups [12].

Our findings are largely consistent with the post-HHFKA analysis, and because of our detailed information on school meal foods, they can provide some additional insights. Nonschool meal foods eaten by students in all food security groups scored well below the optimal score for most components, and the scores were similar across the groups with only two exceptions—dairy and fatty acids. The lower dairy scores for nonschool foods among FI students suggest school meal foods are particularly important for calcium and other essential nutrients contributed by dairy; this is consistent with findings from SNDA-III that school meal participants from FI households obtained 47% of their calcium intake from school meals, compared to 38% of intake for participants from FS households [10]. Although fatty acid scores from non-school foods were higher among FI students, all groups scored less than 50% for this component, suggesting improvement is needed by all.

School foods scored at or close to 100% for five HEI components, including fruit, dairy, and whole grains. For some HEI components, the quality gap between school and nonschool foods was particularly large—for example, nonschool food scores averaged 31% of the HEI score for whole grains, whereas school foods scored 100%. For each student group, the average HEI scores for nonschool foods were between 55% and 57%, whereas school foods scored between 79% and 81%. These findings are consistent with the hypothesis that school meals contribute to students’ dietary quality, but differences across food security groups were not observed. Disappointingly, school food consumption scores for total vegetables, and especially dark green vegetables and beans, were below 50% for all student groups, despite SNMCS-I menu data indicating that meals provided to students scored much higher on these components [26]. Low scores for these foods indicate a challenge for creating acceptance of these foods. Scores for sodium were also low, less than 50%; in this case, menu data indicate some schools found it challenging to meet sodium targets [26]. The USDA has worked to assist SFAs with this challenge by developing low-sodium USDA Foods for schools; other strategies the USDA has identified as effective include using taste tests to identify student preferences, tailoring menu options to cultural and local preferences, and nutrition promotion [27].

A few limitations must be noted. First, students were categorized using the adult measure of food security. Because students’ status may differ from that of the adults in the household, the adult measure may be less sensitive for detecting the contribution of school meals to students’ diets across food security groups. Second, children and adolescents, like adults, may misreport their dietary intakes, either intentionally or because they are still undergoing cognitive development [28]. Several dietary recall validation studies have found children to accurately or slightly overreport their energy intakes, and younger children may report more accurately with parental assistance, but overweight or obese children and older children are more likely to underreport [28,29]. Third, some analyses were based on small sample sizes and may not be reliable; for example, few students attended schools that offered universal free meals. Finally, SNMCS-I was a descriptive study that was not designed to test causality of any hypotheses and the analysis in this paper does not attempt to account for any possible confounders or adjust for characteristics that may be associated with misreporting. The relationships among student, household, or school characteristics, school meal participation, and dietary intakes should not be interpreted as causal.

### Implications for Practice and Future Research

U.S. federal dietary guidance emphasizes the importance of a healthy diet in childhood for growth, development, learning, and establishment of healthy eating habits that may reduce the risk of health problems later in life [30]. For children from FI households, USDA school meals may be particularly important. Our findings support those of previous research indicating that USDA school meals can be important contributors to the diets of children in FI households, augmenting household food resources [8,10,11]. Participation in school meals may also increase acceptance of healthier foods outside of school, similar to what has been found for another USDA-sponsored, school-based child nutrition program [31]. Nevertheless, some children in these households do not participate in school meals, especially SBP. Providing universal free meals was associated with higher participation. The CEP provides a new mechanism for schools serving primarily low-income children to offer universal free meals, and its adoption has grown from 14,214 schools in SY 2014–2015, its first year of national availability, to 30,667 in SY 2019–2020 [32]. Future research will provide more information on the association of CEP adoption with SBP participation among children from FI households. Research on other strategies to promote SBP participation, such as serving it in the classroom at the start of the school day rather than in the cafeteria before school, may also be helpful to school nutrition professionals interested in promoting SBP participation, and research may also shed light on strategies for motivating healthier eating outside of school.

It is notable that school foods promote diet quality among all students, whether food secure or food insecure. Emphasizing this finding may encourage participation among all students, creating a norm that would reduce any perceived stigma among FI students.

## Figures and Tables

**Table 1:** Healthy Eating Index-2010 components and standards for scoring.

	Maximum Score	Standard for Maximum Score ^a^	Standard for Minimum Score of Zero ^a^
Adequacy components: Higher scores reflect higher concentrations in students’ diets			
Total fruit ^b^	5	≥0.8 c equivalent per 1000 kcal	No fruit
Whole fruit ^c^	5	≥0.4 c equivalent per 1000 kcal	No whole fruit
Total vegetables ^d^	5	≥1.1 c equivalent per 1000 kcal	No vegetables
Greens and beans ^d^	5	≥0.2 c equivalent per 1000 kcal	No dark green vegetables, beans, or peas
Whole grains	10	≥1.5 oz equivalent per 1000 kcal	No whole grains
Dairy ^e^	10	≥1.3 c equivalent per 1000 kcal	No dairy
Total protein foods ^f^	5	≥2.5 oz equivalent per 1000 kcal	No protein foods
Seafood and plant proteins ^f,g^	5	≥0.8 oz equivalent per 1000 kcal	No seafood or plant proteins
Fatty acids ^h^	10	(PUFAs ^i^ + MUFAs ^j^)/saturated fatty acids ≥ 2.5	(PUFAs + MUFAs)/saturated fatty acids ≤ 1.2
Moderation components: Higher scores reflect lower concentrations in students’ diets			
Refined grains	10	≤1.8 oz equivalent per 1000 kcal	≥4.3 oz equivalent per 1000 kcal
Sodium	10	≤1.1 g per 1000 kcal	≥2.0 g per 1000 kcal
Empty calories ^k^	20	≤19% of energy	≥50% of energy
Total Score	100		

Adapted from the U.S. Department of Agriculture [20]. ^a^ Concentrations between the minimum and maximum standard are scored proportionately. Higher scores reflect higher nutritional quality. ^b^ Includes 100 percent fruit juice. ^c^ Includes all forms except juice. ^d^ Includes any beans and peas not counted as total protein foods. ^e^ Includes all milk products, such as fluid milk, yogurt, cheese, and fortified soy beverages. ^f^ Beans and peas are included here (and not with vegetables) when the total protein foods standard is otherwise not met. ^g^ Includes seafood, nuts, seeds, soy products (other than beverages) and beans and peas counted toward total protein foods. ^h^ Ratio of polyunsaturated and monounsaturated fatty acids (PUFAs and MUFAs) to saturated fatty acids. ^i^ PUFA = polyunsaturated fatty acid. ^j^ MUFA = monounsaturated fatty acid. ^k^ Kcals from solid fats, added sugars, and alcoholic beverages. Threshold for counting alcohol is >13 g/1000 kcal. c: cup; g: gram; kcal = calorie; oz: ounce.

**Table 2:** Student, household, and school characteristics by food security status ^1^.

	Food Insecure (*n* = 342)		Marginally Secure (*n* = 255)		Food Secure (*n* = 1246)		Total (*n* = 1843)	
	%	95% CI	%	95% CI	%	95% CI	%	95% CI
Student and household characteristics								
Age:								
Less than 9 years	23.3	(16.9, 29.8)	23.9	(16.7, 31.1)	20.2	(16.6, 23.8)	21.2	(17.8, 24.6)
9 to 13 years	42.3	(34.3, 50.4)	40.5	(31.9, 49.2)	38.1	(34.3, 41.9)	39.0	(35.3, 42.7)
Greater than 13 years	34.4	(25.6, 43.1)	35.6	(26.1, 45.1)	41.7	(36.2, 47.3)	39.8	(34.6, 45.0)
Female	53.8	(45.9, 61.7)	45.8	(38.5, 53.1)	48.8	(46.4, 51.3)	49.2	(46.6, 51.7)
Race/ethnicity:								
Hispanic	44.7 ^c^	(35.9, 53.6)	39.2 ^b^	(27.9, 50.4)	18.7 ^bc^	(14.1, 23.3)	25.4	(19.9, 30.9)
White, non-Hispanic	31.4 ^c^	(23.0, 39.8)	34.7 ^b^	(23.9, 45.4)	60.1 ^bc^	(53.4, 66.7)	52.3	(45.7, 58.9)
Black, non-Hispanic	16.7	(9.7, 23.7)	18.2 ^b^	(10.7, 25.7)	11.6 ^b^	(6.3, 16.9)	13.3	(8.2, 18.3)
Other	7.2	(4.0, 10.4)	7.9	(3.1, 12.7)	9.6	(7.3, 11.9)	9.0	(7.2, 10.9)
Poverty level:								
Up to 130% FPL	72.1 ^ac^	(64.8, 79.4)	60.5 ^ab^	(51.8, 69.2)	19.1 ^bc^	(14.7, 23.6)	32.7	(27.6, 37.7)
131 to 185% FPL	15.4 ^c^	(9.5, 21.3)	18.1 ^b^	(12.0, 24.2)	5.9 ^bc^	(4.1, 7.7)	9.0	(7.1, 10.8)
Greater than 185% FPL	12.5 ^ac^	(7.7, 17.2)	21.4 ^ab^	(13.4, 29.3)	75.0 ^bc^	(69.8, 80.2)	58.4	(52.6, 64.2)
FRP-certified	85.2 ^c^	(78.1, 92.2)	79.3 ^b^	(72.1, 86.5)	31.1 ^bc^	(25.1, 37.1)	45.7	(39.3, 52.2)
HEI-2010 score	64.3	(61.2, 67.3)	64.4	(61.7, 66.9)	63.3	(61.7, 64.9)	63.6	(62.3, 64.9)
School characteristics								
Offered SBP	98.4 ^c^	(96.5, 100.3)	98.1 ^b^	(96.2, 100.1)	91.0 ^bc^	(84.2, 97.8)	93.1	(87.7, 98.4)
Offered universal free meals	17.5 ^c^	(6.6, 28.4)	18.2 ^b^	(8.2, 28.2)	8.9 ^bc^	(3.2, 14.6)	11.3	(4.9, 17.6)
Offered reimbursable snacks or suppers	41.7 ^c^	(29.5, 53.8)	38.1 ^b^	(25.8, 50.4)	18.9 ^bc^	(12.1, 25.7)	25.0	(17.1, 32.9)
HEI-2010 score for NSLP lunches prepared	83.0	(82.1, 83.8)	82.2	(81.3, 83.1)	82.0	(81.1, 82.9)	82.2	(81.4, 83.0)
HEI-2010 score for SBP breakfasts prepared ^2^	71.4	(70.1, 72.8)	72.1	(70.7, 73.5)	70.8	(69.7, 71.8)	71.1	(70.0, 72.1)
Student school meal participation								
Participated in NSLP	79.0 ^c^	(74.1, 83.9)	71.4 ^b^	(62.3, 80.5)	48.7 ^bc^	(43.1, 54.3)	56.3	(51.1, 61.5)
Participated in SBP	37.5 ^c^	(29.1, 46.8)	32.8 ^b^	(24.6, 40.9)	16.1 ^bc^	(12.1, 20.1)	21.6	(17.1, 26.0)
Participated in both NSLP and SBP	31.9 ^c^	(24.8, 39.0)	29.6 ^b^	(21.7, 37.5)	13.8 ^bc^	(10.2, 17.4)	18.7	(14.7, 22.6)
Participated in either NSLP or SBP	84.5 ^ac^	(80.5, 88.5)	74.5 ^ab^	(65.6, 83.5)	50.9 ^bc^	(45.1, 56.8)	59.2	(53.7, 64.6)

^1^ Food security status was based on the adult food security measure. ^2^ Among schools that offered SBP. ^a^ *p* < 0.05 for the comparison between the food insecure and marginally secure groups. ^b^ *p* < 0.05 for the comparison between the marginally secure and food secure groups. ^c^ *p* < 0.05 for the comparison between the food secure and food insecure groups. CI, confidence interval; FPL, federal poverty line; FRP, free or reduced-price; HEI, Healthy Eating Index; National School Lunch Program; SBP, School Breakfast Program.

**Table 3:** Target day school meal participation rates by food security status and other student and school characteristics ^1^.

	Food Insecure		Marginally Secure		Food Secure		Total	
	%	95% CI	%	95% CI	%	95% CI	%	95% CI
NSLP participation rates								
Number of students	342		255		1246		1843	
All students	79.0	(74.0, 83.9)	71.4	(62.3, 80.5)	48.7	(43.1, 54.3)	56.3	(51.1, 61.5)
FRP-certified:								
Yes	82.6 *	(77.0, 88.1)	78.7 *	(70.8, 86.6)	75.5 *	(67.8, 83.3)	78.2 *	(72.6, 83.9)
No	57.7	(39.0, 76.5)	41.9	(20.5, 63.2)	36.4	(30.5, 42.3)	37.6	(31.4, 43.7)
Universal free lunch offered:								
Yes	83.4	(73.9, 93.0)	83.1	(70.9, 95.3)	79.2 *	(68.9, 89.4)	80.9 *	(74.2, 87.6)
No	77.8	(71.9, 83.8)	66.1	(53.9, 78.3)	43.2	(37.5, 48.8)	50.4	(44.8, 55.9)
Reimbursable snacks or suppers offered:								
Yes	83.1	(76.6, 89.6)	77.8	(67.7, 87.9)	61.2 *	(51.1, 71.4)	70.2 *	(63.0, 77.3)
No	76.0	(69.2, 82.8)	67.3	(54.2, 80.4)	45.5	(39.2, 51.8)	51.6	(45.5, 57.7)
SBP participation rates among students attending schools participating in SBP								
Number of students	339		250		1146		1735	
All students	38.1	(29.6, 46.5)	33.4	(25.2, 41.6)	17.7	(13.5, 21.8)	23.2	(18.7, 27.6)
FRP-certified:								
Yes	39.4	(30.9, 47.8)	36.9 *	(27.6, 46.2)	35.3 *	(28.3, 42.3)	36.8 *	(30.9, 42.7)
No	31.1	(6.3, 55.9)	16.2	(4.5, 28.0)	8.5	(5.2, 11.8)	9.9	(6.3, 13.5)
Universal free breakfast offered:								
Yes	54.6 *	(41.0, 68.2)	51.1 *	(36.9, 65.3)	43.2 *	(31.3, 55.2)	47.6 *	(39.4, 55.9)
No	28.5	(20.4, 36.6)	22.8	(13.4, 32.2)	11.6	(8.2, 15.0)	15.1	(11.4, 18.8)
Reimbursable snacks or suppers offered:								
Yes	40.2	(24.7, 55.7)	37.2	(26.7, 47.6)	28.1 *	(20.5, 35.8)	33.1 *	(26.0, 40.1)
No	36.5	(27.4, 45.6)	30.8	(19.5, 42.1)	14.6	(10.0, 19.3)	19.3	(14.1, 24.6)

^1^ Food security status was based on the adult food security measure. * *p* < 0.05 for the comparison between the presence and absence of a characteristic within a food security group. CI, confidence interval; FRP, free or reduced-price; National School Lunch Program; SBP, School Breakfast Program.

**Table 4:** Percentage of 24-h energy intakes from NSLP and SBP foods, by food security status ^1^.

	Food Insecure		Marginally Secure		Food Secure		Total	
	%	95% CI	%	95% CI	%	95% CI	%	95% CI
All students (*n* = 1843)	22.3 ^b^	(19.6, 25.0)	19.9 ^a^	(17.2, 22.6)	13.4 ^ab^	(11.7, 15.0)	15.6	(14.1, 17.1)
Participated in either NSLP or SBP (*n* = 1165)	25.2	(22.1, 28.3)	24.1	(21.0, 27.3)	23.1	(21.2, 24.9)	23.7	(22.3, 25.1)
Participated in both NSLP and SBP (*n* = 369)	29.3	(24.9, 33.7)	32.8	(28.0, 37.5)	30.1	(27.2, 32.9)	30.5	(28.4, 32.6)

^1^ Food security status was based on the adult food security measure. ^a^ *p* < 0.05 for the comparison between the marginally secure and food secure groups. ^b^ *p* < 0.05 for the comparison between the food secure and food insecure groups. CI, confidence interval; National School Lunch Program; SBP, School Breakfast Program.

**Table 5:** HEI-2010 total and component scores, expressed as the mean percentage of the maximum score, for all NSLP and SBP foods consumed by school meal participants, by food security status ^1^.

	Food Insecure (*n* = 281)		Marginally Secure (*n* = 196)		Food Secure (*n* = 688)		Total (*n* = 1165)	
	%	95% CI	%	95% CI	%	95% CI	%	95% CI
Total score	79.8	(77.6, 81.9)	81.3	(77.6, 85.5)	79.3	(77.1, 81.3)	80.0	(78.4, 81.5)
Adequacy components (higher scores reflect higher concentrations in students’ diets)								
Total fruit	100.0	(100.0, 100.0)	100.0	(100.0, 100.0)	100.0	(100.0, 100.0)	100.0	(100.0, 100.0)
Whole fruit	100.0	(100.0, 100.0)	100.0	(100.0, 100.0)	100.0	(100.0, 100.0)	100.0	(100.0, 100.0)
Total vegetables	39.8	(28.2, 51.9)	48.3	(34.9, 63.0)	42.3	(36.2, 48.3)	42.6	(37.0, 48.2)
Greens and beans	26.3 ^	(7.4, 45.1)	41.1 ^	(−1.7, 85.2)	13.5^	(4.7, 23.3)	20.2 ^	(9.4, 34.3)
Whole grains	100.0	(100.0, 100.0)	100.0	(100.0, 100.0)	100.0	(100.0, 100.0)	100.0	(100.0, 100.0)
Dairy	100.0	(100.0, 100.0)	100.0	(100.0, 100.0)	100.0	(100.0, 100.0)	100.0	(100.0, 100.0)
Total protein foods	98.0	(85.3, 100.0)	95.6	(74.7, 100.0)	90.0	(78.7, 100.0)	94.6	(83.1, 100.0)
Seafood and plant proteins	26.3	(13.9, 40.6)	28.1 ^	(11.9, 50.4)	41.0	(28.6, 53.0)	36.6	(24.6, 46.4)
Fatty acids	58.8	(44.2, 73.0)	57.1	(38.0, 82.8)	57.0	(44.9, 69.6)	57.3	(46.7, 68.4)
Moderation components (higher scores reflect lower concentrations in students’ diets)								
Refined grains	100.0	(100.0, 100.0)	99.5	(93.7, 100.0)	96.7	(87.5, 100.0)	99.4	(94.7, 100.0)
Sodium	44.2	(33.3, 54.8)	50.0	(38.7, 61.7)	45.6	(38.3, 52.6)	46.1	(39.9, 52.2)
Empty calories	99.9	(99.1, 100.0)	100.0	(100.0, 100.0)	100.0	(99.4, 100.0)	100.0	(100.0, 100.0)

^1^ Food security status was based on the adult food security measure. No significant differences across the food security groups were found. ^ Because the coefficient of variation was large, the point estimate is considered less precise than other estimates. CI, confidence interval; HEI, Healthy Eating Index; National School Lunch Program; SBP, School Breakfast Program.

**Table 6:** HEI-2010 total and component scores, expressed as the mean percentage of the maximum score, for all non-NSLP or SBP foods consumed by school meal participants by food security status ^1^.

	Food Insecure (*n* = 281)		Marginally Secure (*n* = 196)		Food Secure (*n* = 688)		Total (*n* = 1165)	
	%	95% CI	%	95% CI	%	95% CI	%	95% CI
Total score	54.6	(50.5, 58.7)	55.2	(51.2, 59.0)	57.4	(54.2, 60.5)	56.5	(54.1, 58.9)
Adequacy components (higher scores reflect higher concentrations in students’ diets)								
Total fruit	68.4	(56.0, 81.3)	77.4	(59.9, 96.3)	74.9	(65.0, 85.2)	74.0	(65.6, 82.7)
Whole fruit	89.4	(69.0, 100.0)	90.9	(68.9, 100.0)	91.6	(75.9, 100.0)	91.8	(79.0, 100.0)
Total vegetables	42.8	(37.7, 48.8)	49.0	(35.5, 61.6)	45.4	(40.2, 50.6)	45.0	(40.5, 49.7)
Greens and beans	12.9 ^	(4.7, 32.5)	13.5 ^	(4.7, 25.3)	20.5 ^	(9.9, 34.3)	15.3 ^	(9.6, 29.8)
Whole grains	32.3	(24.2, 40.2)	29.4	(22.9, 36.0)	31.3	(24.6, 37.7)	31.2	(26.5, 35.8)
Dairy	68.2 ^a^	(60.4, 76.1)	73.9	(63.0, 85.2)	80.3 ^a^	(73.9, 87.5)	77.0	(72.0, 82.0)
Total protein foods	95.2	(85.3, 100.0)	93.1	(83.7, 100.0)	99.2	(93.9, 100.0)	98.4	(93.2, 100.0)
Seafood and plant proteins	77.9	(57.1, 98.9)	72.0	(42.4, 100.0)	68.1	(52.8, 83.7)	73.2	(60.8, 84.2)
Fatty acids	45.2 ^a^	(38.5, 52.5)	36.5	(27.5, 46.4)	33.5 ^a^	(27.1, 40.3)	36.3	(31.5, 41.4)
Moderation components (higher scores reflect lower concentrations in students’ diets)								
Refined grains	39.9	(31.0, 49.1)	41.9	(27.6, 55.8)	51.4	(44.3, 58.2)	47.4	(41.8, 53.0)
Sodium	45.8	(37.6, 53.8)	46.6	(39.5, 53.3)	47.5	(43.1, 51.9)	47.0	(43.7, 50.3)
Empty calories	60.7	(54.7, 67.0)	62.9	(55.0, 71.4)	65.0	(59.6, 70.2)	63.8	(59.5, 68.0)

^1^ Food security status was based on the adult food security measure. ^ Because the coefficient of variation was large, the point estimate is considered less precise than other estimates. ^a^ *p* < 0.05 for the comparison between the food secure and food insecure groups. CI, confidence interval; HEI, Healthy Eating Index; National School Lunch Program; SBP, School Breakfast Program.

## Data Availability

No applicant.
